# Accuracy of the Conventional Facial Impression Method and Three-Dimensional Auricular Shape Data Obtained Using Extra- and Intraoral Optical Scanners

**DOI:** 10.3390/dj12110354

**Published:** 2024-11-01

**Authors:** Takumi Kasahara, Meiko Oki, Shingo Kamijo, Hidekazu Takahashi

**Affiliations:** 1 Dental Laboratory, Institute of Science Tokyo Hospital, 1-5-45, Yushima, Bunkyo-ku, Tokyo 113-8549, Japan; 180041kasahara.dtec@tmd.ac.jp; 2 Department of Basic Oral Health Engineering, Graduate School, Institute of Science Tokyo (Science Tokyo), 1-5-45, Yushima, Bunkyo-ku, Tokyo 113-8549, Japan; s-kamijoh.itoe@tmd.ac.jp; 3 Department of Oral Biomedical Engineering, Faculty of Dentistry, Institute of Science Tokyo (Science Tokyo), 1-5-45, Yushima, Bunkyo-ku, Tokyo 113-8549, Japan; takahashi.bmoe@tmd.ac.jp

**Keywords:** facial impression, optical scanner, intraoral scanner, scan accuracy, auricular prosthesis

## Abstract

**Background:** Facial impression methods have been used to fabricate auricular prostheses; however, deformation due to the weight of the impression material remains. This study aimed to investigate the accuracy of auricular three-dimensional (3D) data obtained using a 3D optical extraoral scanner and an intraoral scanner compared with that of a conventional facial impression method. **Methods:** Six auricles were digitized using an extraoral scanner with and without a reference board around the auricle and an intraoral scanner. Auricle casts fabricated using auricle impressions were scanned using a laboratory scanner. All obtained data were superimposed, and the 3D deformations of the different methods were evaluated using measurement mapping and root mean square (RMS) values. The length, width, and height of the auricular casts and scanned data were measured and compared with the sizes of the original auricles measured using a caliper. **Results:** Large displacements of the back and margins of the ears were observed, and the RMS values ranged from 0.5–2.4 mm. Larger widths and smaller heights of the scanned and impression data were observed compared to the anthropometric ear data. **Conclusions:** The study concluded that the fabrication of auricular prostheses using optical scanning is clinically acceptable. All examined scanning techniques demonstrated discrepancies within acceptable limits when compared to the conventional facial impression method.

## 1. Introduction

Facial prostheses are applied to patients who cannot undergo surgical reconstruction of facial defects or morphologic deformities caused by trauma, congenital diseases, or tumor resection [1]. Facial disfigurement can lead to negative social and psychological effects [2,3,4]. Atay et al. [4] used the World Health Organization Quality of Life Instrument, Short Form, and reported that patients with facial prostheses had lower scores in overall quality of life, as well as in physical, psychological, social relationships, and environmental health domains, compared to healthy participants. Facial prostheses play a crucial role in improving aesthetics and quality of life, particularly in psychosocial aspects, for patients with defects. Historically, facial prostheses were fabricated from materials such as gold, silver, paper, and linen cloth in the 16th century and vulcanite rubber in the 19th century [1]. However, several issues were noted, including the weight of silver, unpleasant odors from paper and linen, and the rigidity of vulcanite rubber [1]. In the 20th century, polyurethanes and silicone elastomer materials began to be used for facial prostheses. However, polyurethanes presented serious deficiencies, such as inconsistent processing, moisture sensitivity, and poor polymerization [1]. Currently, silicone elastomers are widely used for facial prostheses because of their nontoxicity, dimensional stability, ability to accept various colorants, and ease of manipulation [1]. Maxillofacial prosthodontists and technicians experience the challenge of fabricating facial prostheses that reproduce appropriate three-dimensional (3D) shapes, especially artificial eye positions. Facial prostheses also require duplication and remanufacturing due to discoloration, deterioration, or enlargement of the defect area caused by tumor recurrence over time [1]. The fabrication process of auricular prostheses is particularly complicated and time-consuming, requires a high level of esthetics, and necessitates a skillful technician to reproduce the natural morphology, emulate natural coloring, and determine the 3D position.

Conventionally, facial impression techniques are used to fabricate facial prostheses [1]. The impression materials commonly employed include irreversible hydrocolloid, light-bodied polysulfide, and polyvinyl siloxane [1]. Because of the low flow properties of irreversible hydrocolloids, adding 50% more water improves its flow characteristics and facilitates the impression procedure [1]. To prevent distortion of the underlying tissues and the impression material, the use of a backing, such as quick-setting plaster or a custom tray, is necessary [1]. In current auricular impression approaches, a plastic retaining ring is adapted around the ear, and the impression material is carefully injected around the back of the auricle and poured over the entire auricle. During this procedure, the patient is positioned such that the auricle is parallel to the ground, which places a heavy physical and emotional burden on the patient, as their movement is restricted for a long time. In addition, the weight of the impression material itself causes deformation of the auricle and can lead to severe tissue distortions of approximately 1–3 mm. This makes it impossible to reproduce accurately the natural shape of the patient’s auricle [5]. In recent years, digital technology has been applied to the fabrication of facial prostheses [5,6,7,8,9,10,11,12,13,14,15,16,17]. Using an optical scanner, 3D surface data can be obtained in a short time, and by using mirror images of the healthy side, digital data of the defective side can be easily obtained. By using additive or subtractive manufacturing processes, several trial patterns of the same or modified shape can be fabricated [5,8,9]. The data can also be saved and stored for easy duplication and remanufacturing. Several methods are available for creating 3D surface data. Computed tomography (CT)-based and magnetic resonance imaging (MRI)-based methods have been reported to be more accurate, reflect internal structures, and be more effective than laser scanning methods in fabricating complex auricular prostheses [18,19]. However, owing to problems such as exposure to radiation and metal-induced artifacts, CT and MRI are not appropriate methods to fabricate facial prostheses. Antonacci et al. [20] analyzed the accuracy of different types of facial scanners and found that many scanners had an accuracy of less than 1 mm, making facial scanners a powerful tool. Handheld portable models can be used for chairside optical impressions [7]. This scanner type automatically aligns itself by referencing the overlapping geometry between frames. Tsuchida et al. [21] showed that a handheld scanning time in the typical range of 15.2–42.2 s results in good accuracy. Moreover, intraoral scanners (IOSs) have been introduced to scan auricles [15,16,17] because extraoral scanners do not allow light to reach the interior of complex auricular structures, resulting in missing data [19]. According to Diker et al. [22], several IOSs were used to scan intraoral models, and a comparison of their scanning accuracy showed that the Trios3 had the highest accuracy, with an error of 58.3 ± 5.9 μm. However, intraoral scanning has been reported to cause misalignment, distortion, and deformation of scan data due to the limited scanning range and data compositing by stitching images together [23,24]. Son et al. [24] showed that IOSs have greater deviation at the far end of the scan than laboratory scanners. However, differences in scanner types and measurement methods, including extraoral and intraoral optical scanners, and their accuracy compared with that of conventional methods have not been evaluated in the auricular region.

The present study aimed to investigate the accuracy of auricular morphology obtained using handheld extraoral and intraoral scanners with that obtained using the conventional facial impression method. The extraoral scanner was also examined for differences in measurement accuracy between scans with and without a reference plane. The null hypothesis was that the amount of 3D displacement and the length, width, and height between two points on the auricle using any of these methods would not differ.

## 2. Materials and Methods

### 2.1. Research Subjects

The research subjects were the left and right lateral auricles of three healthy human beings (two men and one woman, mean age 23.0 years) who were fully informed of the study design and provided written informed consent. The experimental protocols were approved by the Institutional Ethics Committee of the Faculty of Dentistry, Tokyo Medical and Dental University, Tokyo, Japan (approval no. D2014-150).

### 2.2. Measurement of 3D Displacement

**Figure 1:**
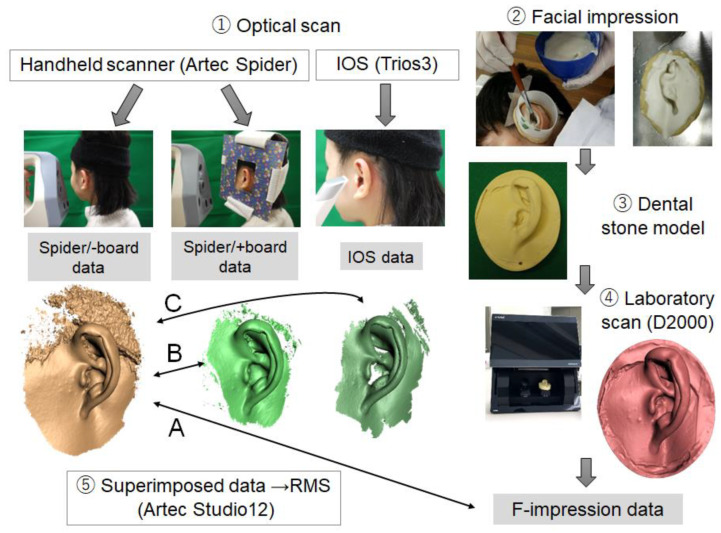
Workflow to determine three-dimensional displacement. Comparison A: Spider/−board and F-impression; Comparison B: Spider/−board and Spider/+board; Comparison C: Spider/−board and IOS. IOS: Trios 3; RMS: root mean square.

To prevent facial deformities caused by the impression-making process, initially optical scans were performed, followed by facial impressions on the same day. The hair was fixed with tape to keep the area around the auricle clear. Optical impressions were obtained in a sitting position, looking slightly downward. For the facial impression, the head was placed on a table so that the auricle was parallel to the floor in a horizontal position. Figure 1 shows the workflow of the 3D displacement measurements.

#### 2.2.1. Optical Impressions

As 3D optical scanners, an extraoral handheld 3D optical scanner (Spider; Artec Spider, Artec Group, Senningerberg, Luxembourg) and an IOS (Trios3, 3Shape, Copenhagen, Denmark) were used in the present study. The 3D point accuracy and resolution of the Artec Spider are 0.05 and 0.1 mm, respectively [25], and the trueness and precision of the Trios3 are 6.9 ± 0.9 µm and 4.5 ± 0.9 µm, respectively [26]. The auricle was scanned in turn with the Spider, followed by the IOS, each with multiple scans.

The scanning sensitivity of the Spider was set to “highest”. As proper processing is not possible without overlapping geometry, placing a patterned board under the object to be scanned increases the number of reference points, allowing for better alignment. Thus, a patterned board as the reference plane was fabricated to be positioned around the auricle as a positioning index. Optical scanning was performed with and without this board, hereafter referred to as Spider/−board and Spider/+board, respectively, and stored as standard triangulated language (STL) data.

Regarding the IOS, the auricle image was obtained by scanning clockwise outward from the auricle in a spiral fashion, followed by a scan of the dorsal surface of the auricle, and stored as STL data, following the method described by Ballo et al. [15].

The obtained STL files were edited to remove unnecessary areas using image editing software (Artec Studio, version 12, Artec Group).

#### 2.2.2. Facial Impression, Model-Making, and Generation of the Morphological Data

Auricular impressions were obtained by a skilled maxillofacial prosthodontist using the conventional facial impression method. A custom-made plastic frame and alginate impression material (Algiace-Z-JP; Dentsply Sirona Inc., Tokyo, Japan) were used. First, the frame was fixed in a position to fit the entire ear, and an alginate mix of 30 g powder with 136 mL ice-water was placed on the auricle using a brush and syringe. Then, a higher concentration alginate mix of 30 g powder with 68 mL ice-water, suggested by the manufacturer, was poured into the frame. After setting the impression material, the impression was removed from the ear and immediately cast with a type 3 dental stone mixture (New Plastone II LE Yellow, GC Corp, Tokyo, Japan).

The stone model of the facial impression was scanned using a laboratory scanner (D2000, 3Shape Dental System version 2.23.1.0, 3Shape), and the image was converted into STL data (hereafter called F-impression).

#### 2.2.3. Analysis Method

Using image editing software (Artec Studio, version 12, Artec Group), each of the two scanned STL files of the same subject were superimposed using the best-fit function.

To evaluate 3D deviation, the root mean square (RMS) value was calculated. The RMS is the numerical value of the error from the overall shape in the ±direction. In this study, measurements were performed ten times by one observer, changing the selected fitting position for each measurement. Because Tsuchida et al. [18] showed that a handheld scan resulted in good accuracy, the reference data were set to the Spider/−board data. The comparisons of the Spider/−board data with the F-impression, Spider/+board, and IOS data were designated as Comparison A, B, and C, respectively (Figure 1). The search distance and display range were set to 10 mm and ±2.00 mm, respectively. Typical displacement distributions were visualized using a mapping display. Mapping display is a function that aligns two data points with respect to the corresponding points on two planes such that the normal vector is the shortest distance between the two data points and displays the normal distance of each part between the two planes as a color map. In regard to the reference data, yellow indicates an elevation of 2.0 mm or more, red indicates an elevation of less than 2.0 mm, green indicates no difference, blue indicates a subsidence of less than 2.0 mm, and light blue indicates a subsidence of 2.0 mm or more.

### 2.3. Comparison of the Auricle Sizes

**Figure 2:**
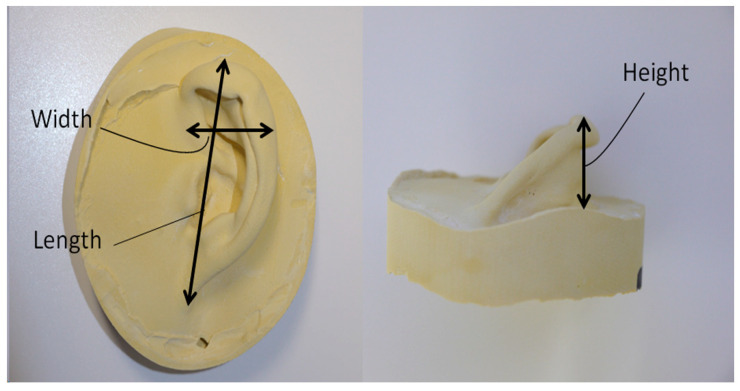
Measured sizes of the auricle—length, width, and height.

The right and left auricles of the three study participants were also directly measured using a caliper (Shinwa Vernier Caliper 19899, Shinwa Rules Co., Ltd., Tsubame, Japan). The maximum permissible error of this caliper is ±0.07 mm. The three measured sizes were length (the distance from the highest part of the auricle to the earlobe), width (the distance of the widest part), and height (the vertical distance of the highest surface from the base; Figure 2). The sizes of the impression models were measured using the caliper, and those of the optical impression STL images were calculated using the image editing software (Artec Studio, version 12, Artec Group). Each data point was measured three times by a single technician.

### 2.4. Statistical Analysis

Data are presented as the mean and standard deviation. Comparisons of 3D displacements (RMS values) of the auricles were statistically analyzed using Wilcoxon’s signed-rank test, as the results of the normality test indicated a lack of normal distribution in all datasets. Regarding auricular sizes, the values directly obtained from the study participants using the caliper were set as the reference, and all other obtained values were statistically examined using the Kolmogorov–Smirnov test for normality, Levene’s test for equality, the Kruskal–Wallis test, and one-way ANOVA. All data were analyzed using SPSS Ver. 27 (IBM, Armonk, NY, USA). Statistical significance was set at *p* < 0.05.

## 3. Results

### 3.1. 3D Displacement

#### 3.1.1. Displacement of the Entire Auricle: RMS Values

**Figure 3:**
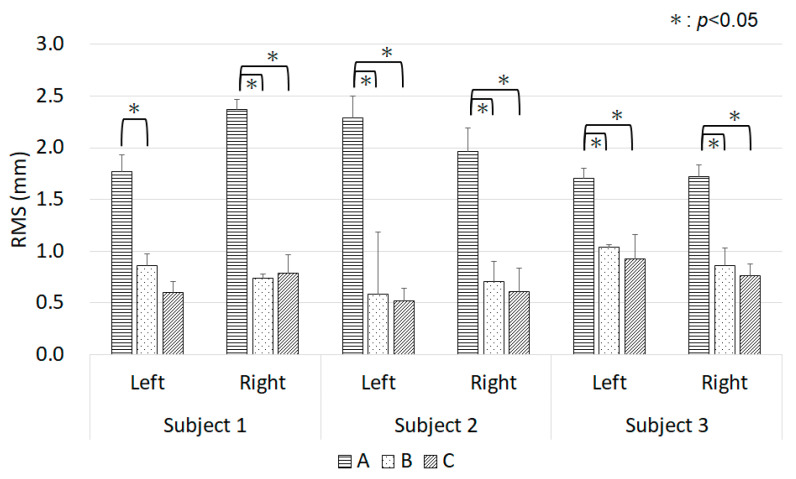
RMS values for Subjects 1–3. Comparison A: Spider/−board and F-impression; Comparison B: Spider/−board and Spider/+board; Comparison C: Spider/−board and IOS. IOS: Trios 3; RMS: root mean square.

Figure 3 shows the RMS values of Comparison A (Spider/−board and F-impression), Comparison B (Spider/−board and Spider/+board), and Comparison C (Spider/−board and IOS). Compared with F-impression, the RMS values of Spider/−board differed by approximately 1.7–2.4 mm. These were the largest differences, as the RMS values differed among the optical impression methods only by approximately 0.5–1.0 mm. The effect of a reference board on Spider measurements (Comparison B) was small (0.59–1.04 mm). The differences between Comparisons A and C and between Comparisons A and B, except for the left auricle of Subject 1 (*p* = 0.076), were significant (*p* < 0.05).

#### 3.1.2. Regional Distribution of the Auricle Displacement

**Figure 4:**
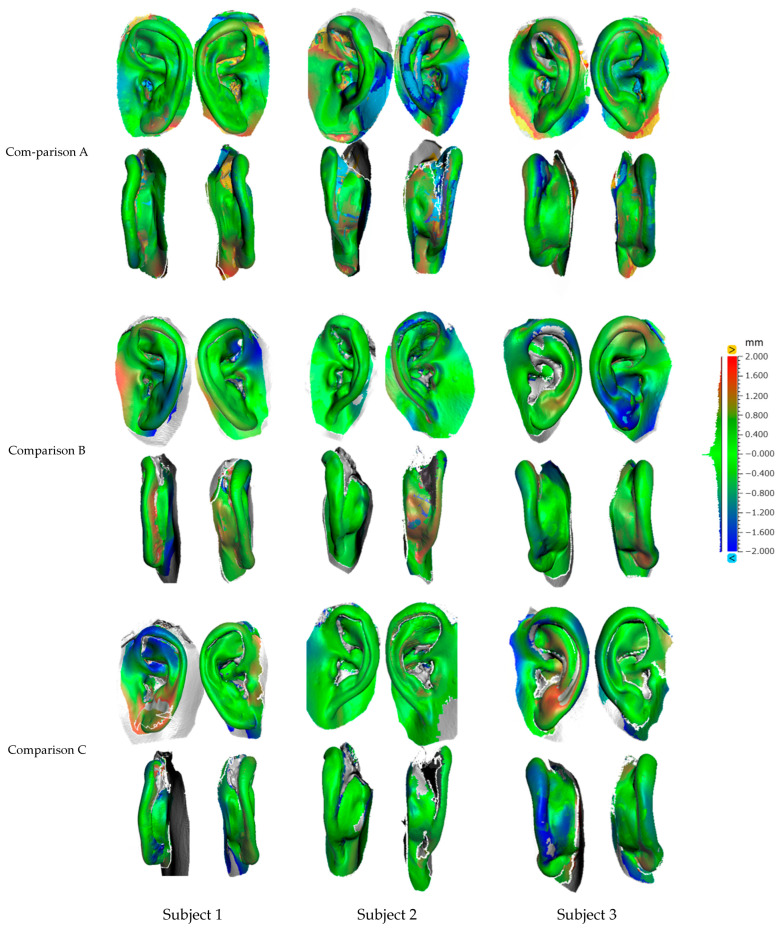
Results of the 3D deviation color maps from Subject 1–3. Comparison A: Spider/−board and F-impression; Comparison B: Spider/−board and Spider/+board; Comparison C: Spider/−board and IOS.

Figure 4 shows color maps of the 3D displacement of the entire auricle of Subjects 1–3. Some areas, such as the deep interior and the base of the dorsal surface of the auricle, could not be scanned with the optical impression methods. With the IOS, it was also difficult to avoid contact with the auricle by the IOS scan head and to capture the dorsal surface of the auricle when the auricular height was high.

In Subject 3, who had a low auricular height, the displacements of the dorsal surface and limbus of the auricle were greater with the Spider/+board than with the Spider/−board. The RMS values varied among subjects by approximately 2.0 mm. In Comparison A, 3D differences were observed on the dorsal surface and limbus of the auricle. In Comparison B, using a reference board led to small differences in the dorsal surface and the height of the auricle. In Comparison C, IOS tended to have a slightly lower auricular height than Spider/−board.

### 3.2. Comparison of the Auricle Sizes

**Figure 5:**
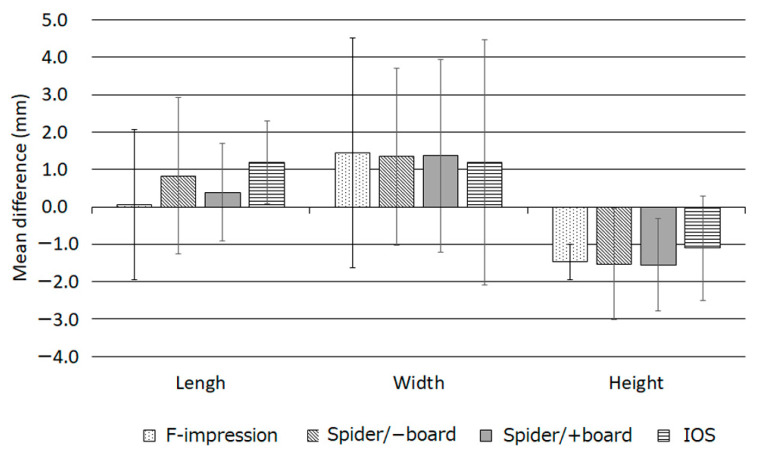
Differences in directly measured auricular sizes. Data are presented as the mean and standard deviation.

The mean values of length, width, and height of Subjects 1–3 are listed in Table 1. Generally, a large variation in the measured values was observed. Using the sizes directly measured with the caliper as the reference, the length was increased by up to 4.6 mm and 3.9 mm for F-impression and Spider/−board, respectively, and the width was up to 7.72 mm larger for IOS, but no significant difference was found using one-way ANOVA (*p* = 0.77). The height was up to −3.9 mm, smaller than that in direct measurements. Figure 5 shows the differences between directly measured sizes of the auricle and sizes measured using other methods. The lengths and widths were slightly increased, whereas the heights were generally reduced.

## 4. Discussion

This comparative study on the accuracy of auricular morphology obtained using facial impressions and extraoral and intraoral scanners rejected the null hypothesis that the amount of 3D displacement would not differ among the examined methods. Instead, significant differences in auricular sizes and variations in the accuracy of the scanning methods were observed. However, a discrepancy of 5 mm in length and 3–4 mm in width in a normal auricle is not considered clinically significant [27]. The accuracies of the examined methods were within these values and, therefore, considered clinically acceptable. Although the number of subjects was small (6 auricles), the present research could determine whether the auricle morphology significantly differed depending on the employed method in the clinical situation.

Regarding the accuracy of facial scanners, Antonacci et al. [20] reported an average difference from direct anthropometry of approximately 1.10–1.74 mm. The optical scanner data in this study differed by 1.7–2.4 mm from the F-impression data, which is clinically acceptable for the fabrication of auricular prostheses. Differences between either scanning method and F-impression cannot be visually recognized, and thus, these methods are applicable for fabricating auricular prostheses. Coward et al. [18] compared auricular sizes obtained from direct MRI scans of a natural auricle with those from CT and laser scans of a cast made using conventional methods. Although the laser scan was less accurate than CT and MRI, the three methods did not significantly differ. Moreover, Coward et al. [19] compared auricular differences between scanned and actual measurements using CT, MRI, and laser scanners. The length, width, and height differences were in the range of 0.18–0.26, 0.01–0.67, and 0.23–0.67 mm, respectively, with CT having the highest accuracy, MRI being within acceptable limits, and laser scans having limitations regarding the internal form. Emam et al. [28] studied the effect of different IOSs and post-space depths on the trueness of digital impressions and concluded that there were significant differences in the trueness of IOSs. In our study, between-subject differences of the internal and dorsal auricular regions were observed, suggesting that compared to CT and MRI, optical impressions are unfavorable in regions with large undercuts. However, length, width, and height did not significantly differ for any of the examined methods. The RMS difference of the F-impression model was 1.7–2.4 mm larger than that of the optical impression methods with 0.5–1.0 mm. This indicates that the conventional method induced significant deformation owing to the weight of the impression materials, making optical impression methods more favorable.

A comparison of the mean RMS values showed that the optical impression data varied among subjects. Subject 1 had a low auricular height (14.4–14.7 mm), which may have prevented the scanner light from reaching deep into the auricle, resulting in lower scanning accuracy. Subjects 2 and 3 had higher auricular height values (18.4–21.1 mm); therefore, the back surfaces of their auricles could be scanned more clearly than that of Subject 1. This resulted in a larger number of areas with displacements of ≤0.8 mm. However, scanning the back surface of the auricle without touching it was difficult with the IOS because a high auricular height requires large movements of the IOS head. A technical report by Ballo et al. [15] showed a digital scanning pattern using IOS but did not compare scan data with actual auricle measurements. The results of the present study suggest that although both ear shape and dimensions can be scanned within 3 mm RMS compared to the F-impression, the height, angle, size, and morphology of the auricle should be considered in the future.

For the extraoral Spider scanner, a board was used as a reference plane to facilitate positioning. This board decreased the error in some subjects and increased it in others. When the auricle height is low, the board interferes with the scanner light; thus, scanning the back surface of the auricle and the margin may become difficult. However, in subjects with high auricular height, the use of this reference board made no difference. This suggests that morphological factors such as the tilt and height of the auricle are more important than the presence of a reference board. The image acquisition time, an important aspect of clinical applications, was not recorded in our study. This factor should be considered in future studies, as the scanning time might be shortened by the presence of a board, as it creates a reference plane.

In our preliminary study using the standard scan sensitivity of the Artec Spider, the obtained data were generally smaller, and the RMS values showed deformations of 2.19–3.28 mm. Using the highest sensitivity setting in the present study improved the RMS values, ranging from 0.59 to 2.68 mm. Therefore, increasing the scanner sensitivity reduces the variation in RMS values and improves scan accuracy. However, scanning with high sensitivity also acquires a large amount of unwanted noise data, and great care should be taken when editing the data from the margins and interior of the ear.

An optical scanner stitches images together to combine them. Data distortion has been reported to increase further away from the starting point of the measurement in scanning dentition [23,24]. In the present study, a comparison of the auricular surface sizes showed a slight increase in width and a slightly lower overall height for all methods. Thus, the thin apex of the helix cannot be scanned properly as a 3D object in the optical impression method, and deformation owing to the weight of the impression material occurs in the facial impression method. Regarding height, many areas could not be scanned optically, which may have caused distortion when combining data from optical impressions. Regarding the IOS, the ease with which the back of the auricular surface can be removed varies greatly from person to person, which may result in a lower height. Regarding the Artec Spider, the reference board might prevent distortions during data synthesis, but the mean differences did not improve. In this study, a hard flat board was used, and the scanner light was sometimes obstructed. It will be necessary to consider more accurate scanning methods in the future, such as setting up a reference surface that allows the light to reach all regions around the ear.

Facial prostheses must appear natural and should preferably match the residual face with symmetry [29]. However, perfect facial symmetry does not exist, and generally, a difference of 3 to 4 mm or less is not perceived as asymmetrical [30]. In the present study, the comparison of the two-dimensional accuracy of the ears showed that most of the scan data differed from the actual measurements by 4 mm or less, suggesting that this level of discrepancy should not pose any issues in clinical applications. 

Unkovskiy et al. [17] reported a method to fabricate an auricular prosthesis directly by material extrusion using a silicone elastomer; however, the margin area did not match the computer-assisted design data; therefore, a final conventional step was needed to coat the prosthesis with silicone material. In the present study, only the scannable surface of the auricle was digitized. However, to design an auricular prosthesis completely on a computer, further studies are necessary to examine the data accuracy of the marginal area, compare it with the 3D construction based on CT images, and measure reflecting areas, such as the skin and mucosa at the auricular defect. Because auricular morphology is complex and some ears in this study were difficult to scan, future studies should be performed to examine the differences due to factors such as auricular morphology, contour, and internal structure. Additionally, the accuracy of the shape and fit of the auricular pattern formed by the 3D modeling machine should be evaluated to determine whether this approach can be applied to the fabrication of auricular prostheses.

In the present study, some limitations were identified. First, the number of scanned auricles was limited to six, and the reference data were not obtained from accurate original in vivo measurements. Furthermore, some optical data were missing in the deep and complex areas of the auricles when using optical scanners. Further studies are needed to increase the sample size, employ the latest high-accuracy scanners, and compare the results with facial reference data obtained from CT or MRI.

## 5. Conclusions

Based on the comparative study of data obtained by scanning models made using the facial impression method with a laboratory scanner and shape data obtained using two different optical impression methods (a 3D non-contact handheld scanner and an IOS), the following conclusions were drawn:

1. Optical impression methods cannot scan some areas of the auricle.
2. All optical impressions had lower RMS values than F-impression and can be considered clinically acceptable for auricular prostheses fabrication.
3. Using the Artec Spider, differences in RMS values of scans with and without the reference board were less than 1.04 mm.
4. Both facial and optical impressions were scanned with slightly larger widths and lower heights than the actual auricular dimensions because of deformations caused by the weight of the impression material and distortions caused by image stitching.
5. Significant differences in auricular sizes and variations in the accuracy of the scanning methods were observed, with discrepancies within 3 mm in length. However, these discrepancies were considered clinically acceptable.

## Figures and Tables

**Table 1:** Distance between two points of length, width, and height of the auricle.

		Subject 1		Subject 2		Subject 3	
		Left	Right	Left	Right	Left	Right
Length	Direct	58.47 ± 0.57	61.20 ± 0.10	61.30 ± 0.20	63.40 ± 1.15	59.00 ± 0.20	59.20 ± 1.06
	F-impression	63.10 ± 0.20	61.80 ± 0.26	63.10 ± 0.44	61.93 ± 0.50	59.37 ± 0.31	60.37 ± 0.49
	Spider/−board	62.37 ± 0.29	61.06 ± 0.22	62.73 ± 0.56	61.10 ± 0.16	58.50 ± 0.47	59.16 ± 0.04
	Spider/+board	60.74 ± 0.97	62.02 ± 0.42	63.82 ± 0.21	63.34 ± 0.28	59.01 ± 0.24	58.62 ± 0.33
	IOS	59.87 ± 0.16	60.50 ± 0.45	62.86 ± 0.08	62.59 ± 0.67	58.46 ± 0.56	58.61 ± 0.34
Width	Direct	26.17 ± 0.45	29.40 ± 0.61	30.47 ± 0.55	29.60 ± 0.62	31.50 ± 0.60	32.90 ± 0.44
	F-impression	32.43 ± 0.35	31.07 ± 0.74	27.50 ± 0.56	31.57 ± 0.15	32.27 ± 0.06	32.30 ± 0.50
	Spider/−board	31.28 ± 1.00	28.68 ± 0.21	28.85 ± 0.30	31.47 ± 0.64	33.03 ± 1.02	34.95 ± 0.21
	Spider/+board	31.58 ± 0.06	32.13 ± 0.94	28.33 ± 0.43	29.84 ± 0.26	33.17 ± 0.21	33.06 ± 0.13
	IOS	33.89 ± 0.36	29.27 ± 0.39	30.00 ± 0.74	30.53 ± 0.18	33.40 ± 0.57	31.55 ± 1.28
Height	Direct	14.70 ± 0.20	14.40 ± 0.40	21.07 ± 0.61	21.10 ± 0.62	19.70 ± 0.10	18.43 ± 0.15
	F-impression	14.17 ± 0.23	13.33 ± 0.49	20.10 ± 1.68	19.63 ± 0.67	17.90 ± 0.72	17.67 ± 0.32
	Spider/−board	13.64 ± 0.57	13.18 ± 0.36	18.41 ± 0.47	17.19 ± 0.32	19.95 ± 0.21	17.75 ± 0.21
	Spider/+board	13.60 ± 0.18	12.29 ± 0.38	18.65 ± 0.24	18.13 ± 0.59	20.16 ± 0.27	17.38 ± 0.56
	IOS	13.64 ± 0.24	14.68 ± 0.26	19.51 ± 0.44	17.57 ± 0.85	19.26 ± 0.52	15.87 ± 0.67

Data are presented as the mean ± standard deviation in mm. Direct: direct measurement of the auricle; F-impression: measurement of the model made from facial impressions; IOS: Trios 3 data; Spider/−board: Artec Spider data without the reference board; Spider/+board: Artec Spider data with the reference board. There were no significant differences among these data using one-way ANOVA.

## Data Availability

The data presented in this study are available at the request of the corresponding author for ethical reasons.
